# Perioperative Management of a Pregnant Patient With Severe Mitral Stenosis and Pulmonary Hypertension for Repeat Cesarean Section: The Role of Standby Extracorporeal Membrane Oxygenation

**DOI:** 10.7759/cureus.89986

**Published:** 2025-08-13

**Authors:** Avaish Kachhwaha, Daniela S Karagyozyan, Nadia Aluzri, Rashmi Vandse

**Affiliations:** 1 Anesthesiology and Perioperative Medicine, Loma Linda University Medical Center, Loma Linda, USA; 2 Anesthesiology and Perioperative Medicine, Loma Linda University School of Medicine, Loma Linda, USA

**Keywords:** cesarean delivery, high-risk pregnancy, maternal cardiac disease, mitral valve stenosis, obstetric anesthesia, pulmonary hypertension in pregnancy, va-ecmo

## Abstract

Managing pregnant patients with severe mitral stenosis (MS) and pulmonary hypertension (PH) remains a challenge, requiring a multidisciplinary approach to mitigate maternal and fetal risks. We report a case of a 29-year-old female, gravida 6 para 5, at 37 weeks’ gestation with severe rheumatic MS, significant PH, and right ventricular (RV) dysfunction who underwent an elective repeat cesarean delivery. Due to her elevated risk of hemodynamic collapse, invasive monitoring, incremental epidural anesthesia, and standby extracorporeal membrane oxygenation (ECMO) were utilized. Intraoperative vasopressor and uterotonic selection was optimized to maintain systemic perfusion while minimizing pulmonary vascular resistance. The procedure was successfully completed without complications, with close inpatient monitoring post-partum; both maternal and neonatal outcomes were favorable. This case highlights the value of detailed perioperative preparation, multidisciplinary coordination, and personalized anesthetic and pharmacologic management in pregnancies complicated by severe cardiac disease.

## Introduction

Cardiovascular disease remains a leading cause of maternal morbidity and mortality in the United States, with pulmonary hypertension (PH) and severe mitral stenosis (MS) among the most critical cardiac conditions encountered during pregnancy [1,2]. Both are considered high-risk conditions, with severe MS designated as a World Health Organization (WHO) Class IV condition - indicating that pregnancy is contraindicated due to significantly elevated maternal morbidity and mortality [1]. Despite this, pregnancies in women with severe MS and PH continue to occur and are rising, likely reflecting advances in diagnosis, multidisciplinary care, and therapeutic options [3].

Although rheumatic heart disease - the primary cause of MS - is more common in developing nations, it remains a concern in underserved US populations, where delayed diagnosis can result in complex presentations [4]. Severe MS increases left atrial (LA) pressure and pulmonary venous congestion, often resulting in PH classified as Group 2 PH - secondary to left heart disease. This subtype of PH carries among the highest maternal cardiovascular complication rates [5]. Indeed, the maternal major adverse cardiovascular event (MACE) rate in PH has been reported at up to 25%, compared to under 0.5% in patients without cardiac disease [6].

Given these substantial risks, anesthesiologists must remain well-versed in the evolving management of pregnant patients with complex cardiac disease. A thorough understanding of cardiovascular physiology, hemodynamic targets, and risk stratification is essential to optimize perioperative maternal outcomes. In particularly high-risk scenarios, proactive planning - including the standby availability of extracorporeal membrane oxygenation (ECMO) - serves as a critical safety measure should decompensation occur during induction, delivery, or the immediate postpartum recovery.

We present the case of a pregnant patient with concurrent severe MS and Group 2 PH, detailing our multidisciplinary perioperative strategy with an emphasis on anesthetic considerations and ECMO readiness. This case highlights the critical role of anesthesiologists in managing maternal hemodynamics and underscores the value of proactive planning in complex, high-risk cardiac pregnancies.

## Case presentation

**Figure 1:**
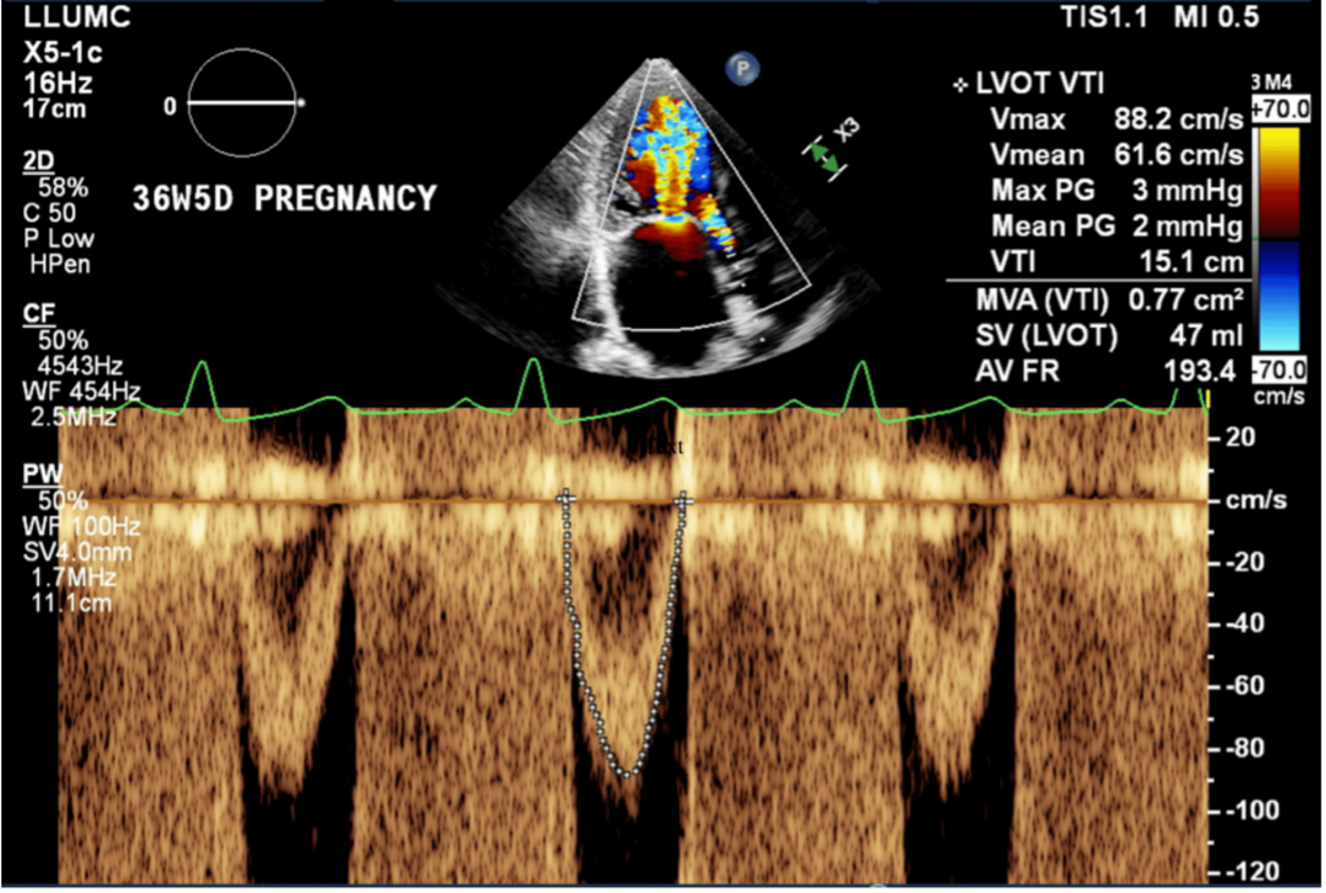
Doppler echocardiographic assessment of the left ventricular outflow and mitral valve area at 36 weeks' gestation Continuous-wave Doppler echocardiography demonstrating the left ventricular outflow tract (LVOT) velocity-time integral (VTI) in a 36-week pregnant patient with severe rheumatic mitral stenosis. The calculated MVA via VTI is 0.77 cm², consistent with severe stenosis. LVOT VTI is 15.1 cm, with a stroke volume of 47 mL and an AV FR of 193.4 mL/s. Abbreviations: LVOT = left ventricular outflow tract; VTI = velocity-time integral; MVA = mitral valve area; SV = stroke volume; AV FR = aortic valve flow rate; PG = pressure gradient

A 29-year-old woman (BMI: 34 kg/m²), G6P5 at 37 weeks' gestation, was admitted for a planned induction of labor. She had a history of rheumatic heart disease with severe MS (mean transmitral gradient: 18 mmHg, peak gradient: 29 mmHg on echocardiography), accompanied by severe PH (right ventricular (RV) systolic pressure (RVSP) of 82 mmHg) and moderate-to-severe RV dysfunction. Despite disease progression since her last pregnancy, she remained asymptomatic (metabolic equivalents (METs): > 4) and was not taking any medications. Her obstetric history included four vaginal deliveries and a cesarean section two years prior to the current presentation. At 33 weeks, she was evaluated by cardiology for possible valvuloplasty; however, given her lack of symptoms, the team recommended deferring mitral valve repair until after delivery. Apart from a gravid abdomen and a cardiac murmur, her physical examination - including fetal assessment - and admission laboratory results were unremarkable. Key echocardiographic findings are summarized in Table 1, with a detailed view of the mitral valve shown in Figure 1.

**Table 1:** Echocardiographic findings demonstrating severe mitral stenosis and pulmonary hypertension Echocardiographic evaluation of a pregnant patient with rheumatic mitral valve disease prior to cesarean delivery. Findings include severe mitral stenosis with commissural fusion and a mean transmitral gradient of 18 mmHg, an elevated estimated pulmonary artery systolic pressure with an RVSP of 82 mmHg, and right heart dilation with reduced systolic function. Abbreviations: EF = ejection fraction; MV = mitral valve; E velocity = early diastolic transmitral flow velocity; RVSP = right ventricular systolic pressure (estimate of pulmonary artery systolic pressure); TR = tricuspid regurgitation

Echocardiographic Findings	Details
Mitral Valve	Severe stenosis, moderate subvalvular thickening, commissural fusion, MV E velocity 2.4 m/s, mean gradient 18 mmHg (HR 94 bpm), mild to moderate mitral regurgitation
Left Atrium	Severely dilated
Right Atrium	Mildly dilated
Right Ventricle	Moderately dilated, moderate-severely reduced systolic function
Pulmonary Hypertension	RVSP 82 mmHg, TR velocity 4.44 m/s
Left Ventricle	Normal size, borderline low EF (EF 50%)
Septal Motion	Flattening (suggestive of pressure overload)
Aortic, Tricuspid, Pulmonic Valves	Mild to moderate regurgitation (rheumatic disease)
Pericardial Effusion	None detected

A multidisciplinary team - including obstetrics, anesthesia, advanced heart failure cardiology, and cardiothoracic surgery (CTS) - convened to determine the safest delivery strategy. Although induction with forceps-assisted vaginal delivery was initially considered, the team ultimately concluded that her fixed mitral valve obstruction, worsening PH, progressive RV dysfunction, and history of prior cesarean delivery warranted a more controlled approach. Compared to her previous echocardiogram - showing a mean transmitral gradient of 15 mmHg, RV systolic pressure (RVSP) of 50 mmHg, moderate RV dilation, and mildly reduced RV systolic function - her current evaluation revealed notable deterioration, including a mean transmitral gradient of 18 mmHg, RVSP of 82 mmHg, and moderate-to-severe RV systolic dysfunction. Although RV cavity size was unchanged, the marked increase in pulmonary pressures and decline in RV contractility led the multidisciplinary team to classify her peripartum risk as extremely high. Consequently, a planned cesarean delivery was scheduled in the operating room, with ECMO on standby for rapid intervention in case of hemodynamic decompensation.

A labor epidural was placed uneventfully. The standard American Society of Anesthesiologists (ASA) monitors, a radial arterial line, and a right internal jugular central line were secured in the OR. The CTS team established femoral artery and vein access, remaining scrubbed throughout the cesarean delivery with ECMO on standby. The patient received 2 mg midazolam for anxiolysis, and an anesthesia provider provided continuous verbal reassurance. She remained calm, though her heart rate intermittently increased to 100-110 beats per minute with stable blood pressure.

Epidural anesthesia was carefully titrated using a test dose of 100 mcg fentanyl and 2 mL of 2% plain lidocaine, followed by incremental boluses of 3% chloroprocaine in 1-2 mL doses, totaling 5 mL over 19 minutes to achieve a T6 sensory level prior to skin incision. Vital signs and anesthetic level were assessed between boluses to ensure hemodynamic stability. Following the initial epidural dosing, the patient experienced a brief systolic blood pressure drop to the 80s, which was promptly treated with two boluses of 1 unit of vasopressin. A norepinephrine infusion was initiated at 8 mcg/min and titrated down to 3 mcg/min after starting a vasopressin infusion at 0.02 units/min, maintaining stable blood pressure for the remainder of the procedure. After delivery, a slow oxytocin infusion ensured adequate uterine tone. Estimated blood loss was 800 mL. A pulmonary artery catheter floated postpartum revealed pulmonary artery systolic pressures of 100-110 mmHg. For postoperative analgesia, 3 mg of epidural morphine was administered.

The patient had an otherwise uneventful cesarean delivery and was transferred to the coronary care unit (CCU), where ECMO standby was discontinued, and norepinephrine and vasopressin infusions were weaned off. She was initiated on metoprolol BID and monitored in the CCU for 48 hours. After demonstrating sustained hemodynamic stability, all invasive lines were removed, and she was stepped down from intensive care. She was discharged on hospital day four with a plan for outpatient CTS follow-up for mitral valve repair.

The infant required no intervention after delivery and was admitted to the neonatal intensive care unit for monitoring, where she demonstrated stable vital signs, normal feeding, adequate urine and stool output, and an unremarkable examination.

## Discussion

Hemodynamic challenges of labor and delivery

Pregnancy induces profound cardiovascular adaptations to meet the metabolic demands of the fetus. Systemic vasodilation reduces systemic vascular resistance (SVR) by up to 40% from baseline, peaking in the second trimester and persisting until postpartum [7]. Cardiac output (CO) rises by approximately 45%, driven by increases in both heart rate (HR) and stroke volume, and is further augmented by the 40-50% expansion in total blood volume, particularly during labor and the early postpartum period [7].

In MS, the fixed obstruction to LA emptying elevates LA pressure, promotes atrial dilation and hypertrophy, and substantially increases the risk of atrial fibrillation [8]. The pregnancy-related rise in blood volume and CO further elevates LA pressure, and any tachycardia shortens diastolic filling, accelerating transvalvular flow and precipitating pulmonary congestion. PH compounds this burden by increasing pulmonary vascular resistance (PVR) and RV afterload. While initially compensated by RV hypertrophy, sustained afterload can lead to RV dysfunction and eventual failure [2].

Labor and delivery carry the highest hemodynamic risk. Neuraxial anesthesia, oxytocin, or hemorrhage can abruptly lower SVR, compromising perfusion in the setting of limited cardiac reserve. The Valsalva maneuver during the second stage reduces preload and diastolic filling, further raising LA pressure and risking pulmonary edema. Conversely, autotransfusion from uterine contractions, placental delivery, and the immediate postpartum period increases preload sharply. In MS, this preload surge cannot be accommodated, leading to acute LA pressure spikes, worsening PH, and RV decompensation. Hemodynamic shifts continue in the postpartum period, particularly within the first 24-72 hours, when marked increases in CO, preload, and SVR place patients - especially those with valvular lesions - at the greatest risk for symptomatic heart failure and potential circulatory collapse [9].

Given these pathophysiologic challenges, careful control of preload, afterload, and HR is essential. Continuous hemodynamic monitoring and tailored anesthetic management, coordinated by a multidisciplinary pregnancy heart team as recommended by ACOG Practice Bulletin 212 [1], is critical to optimizing maternal outcomes.

Neuraxial vs general anesthesia 

Neuroaxial anesthesia is preferred for high-risk cardiac patients due to its ability to reduce hemodynamic stress and lower mortality compared to general anesthesia [10]. General anesthesia, by contrast, can trigger sympathetic activation, hypoxemia, and hypercapnia; when coupled with the adverse effects of positive pressure ventilation, these changes can markedly impair pulmonary hemodynamics, increasing the risk of acute RV decompensation and cardiac arrest [11]. Among neuroaxial techniques, single-shot spinal anesthesia is generally avoided due to its abrupt hemodynamic effects. Instead, epidural anesthesia with carefully titrated incremental dosing is preferred, as it provides greater hemodynamic stability [10]. Given these patients’ high risk for pulmonary edema, routine prophylactic fluid boluses prior to neuraxial anesthesia should generally be avoided; however, small, judicious boluses may be administered with close hemodynamic monitoring if hypotension develops [12]. Finally, special caution is necessary with test doses containing epinephrine, as this can significantly increase heart rate and worsen pulmonary edema, particularly in symptomatic patients [10].

Vaginal vs cesarean section 

There is no definitive evidence favoring vaginal or cesarean delivery in patients with severe MS and PH, although vaginal delivery is usually preferred. Cesarean sections are typically reserved for obstetric indications or for patients at high risk of decompensation during labor [10,12].

Forceps-assisted vaginal delivery is often preferred in cardiac patients to minimize Valsalva-induced preload reduction and hemodynamic instability [13]. However, in the setting of severe MS with PH and RV dysfunction, the combination of fixed CO and an inability to tolerate labor-associated volume shifts can result in decompensation despite a shortened second stage. Additionally, this patient’s prior cesarean section increased the risks associated with a trial of labor, as uterine rupture or emergent surgical intervention in the setting of maternal hemodynamic decompensation would carry substantial risk. While cesarean delivery carries higher risks - including bleeding, uterine atony, and a two- to fourfold increased risk of venous thromboembolism [14] - it offered a safer, more controlled environment for this high-risk patient, particularly with ECMO standby available to mitigate catastrophic deterioration. ECMO standby significantly improved the safety profile, with maternal cardiac arrest data showing an 87.7% resuscitation success rate with ECMO compared to 58.9% without it [15].

While this setup significantly improved safety, it also posed challenges, as invasive monitoring lines (central and ECMO lines) had to be placed while the patient was awake. To minimize discomfort and anxiety, midazolam was administered, and continuous verbal reassurance was provided. To reduce intraoperative risks, PA catheter floating was delayed until after delivery to minimize arrhythmias and potential decompensation.

Vasopressor management

Norepinephrine and vasopressin are commonly used vasopressors in patients with PH and RV dysfunction due to their ability to support SVR with relatively minimal impact on PVR [16-18]. In this case, vasopressin was prioritized for its pulmonary-sparing properties, with low-dose norepinephrine added to augment SVR as needed. By carefully titrating both agents in tandem, their cumulative doses were kept low, achieving maternal hemodynamic goals while minimizing the risk of RV strain. It is important to note that higher doses of vasopressin carry the potential for coronary vasoconstriction and subsequent RV ischemia [18]. In contrast, phenylephrine should be used cautiously given its strong α-adrenergic agonism, which can increase PVR and worsen RV afterload, while epinephrine’s prominent β-adrenergic activity increases the risk of tachycardia and arrhythmias, potentially destabilizing patients with severe MS [18,19]. For these reasons, neither is preferred as a first-line agent in this population.

Uterotonic management

Carboprost and methergine should be avoided in obstetric patients with MS and PH due to their potential to elevate PVR and impair RV function [20]. Safer uterotonic alternatives include oxytocin and misoprostol; however, rapid intravenous boluses of oxytocin can cause peripheral vasodilation, hypotension, and reflex tachycardia, potentially triggering hemodynamic instability. Therefore, careful dosing is essential, typically beginning with a low initial bolus followed by a slow infusion rate - an approach endorsed by the European Society of Cardiology guidelines for managing obstetric patients with heart disease [21].

Postoperative care

The postpartum period, particularly within the first 24-72 hours, represents a critical window of heightened risk for cardiac decompensation, as rapid hemodynamic shifts and autotransfusion of uterine blood may precipitate RV failure, arrhythmias, and circulatory collapse [9,10,18]. During the early postpartum period, the primary treatment objectives include preventing volume overload, stabilizing systemic blood pressure, and closely monitoring right atrial pressure and overall cardiac function. Oral opioids can cause constipation, increasing intrathoracic pressure during straining and exacerbating PH, highlighting the need for a bowel regimen [20].

## Conclusions

Pregnancy complicated by severe MS and PH carries significant maternal and fetal risks, necessitating early multidisciplinary planning and coordination. In this case, epidural anesthesia was carefully titrated to maintain hemodynamic stability, while proactive preparations - including standby ECMO with pre-emptive cannulation and immediate availability of CTS support - were implemented to address potential cardiovascular collapse. Vasopressor and uterotonic regimens were selected to support systemic perfusion while minimizing increases in pulmonary vascular resistance. Vigilant postpartum monitoring was essential, given the heightened risk of clinical deterioration, particularly in the first 24-72 hours after delivery.

As more women with complex cardiac conditions reach reproductive age, advanced perioperative strategies will increasingly shape obstetric care in specialized centers. This case highlights the benefit of a rarely reported approach in obstetric anesthesia: early integration of ECMO support with a prepared perfusion and surgical team. Our experience underscores how proactive, coordinated measures can mitigate catastrophic outcomes and provides a valuable model for quaternary institutions managing similarly high-risk pregnancies.
